# Prefrontal Aβ pathology influencing the pathway from apathy to cognitive decline in non-dementia elderly

**DOI:** 10.1038/s41398-021-01653-8

**Published:** 2021-10-18

**Authors:** Lin Sun, Wei Li, Guanjun Li, Shifu Xiao, Alzheimer’s Disease Neuroimaging Initiative

**Affiliations:** Alzheimer’s Disease and Related Disorders Center, Department of Geriatric Psychiatry, Shanghai Mental Health Center, Shanghai Jiao Tong University School of Medicine, Shanghai, China

**Keywords:** Human behaviour, Diagnostic markers

## Abstract

The purpose of this study is to investigate the complex connection between apathy and cognitive decline that remains unclear. A total of 1057 non-dementia elderly from the Alzheimer’s Disease Neuroimaging Initiative (ADNI) database received up to 13 years of follow-up and were divided into an apathy negative (−) group of 943 participants and an apathy positive (+) group of 114 participants through the Neuropsychiatric Inventory (NPI)-apathy subitem. Cerebrospinal fluid (CSF) AD biomarkers and amyloid β (Aβ) PET were measured, and their longitudinal changes were assessed using linear mixed-effects models. Risk factors for cognitive decline and apathy conversion were explored through the Cox proportional hazards model. Mediation effects of Aβ pathology on cognition were investigated using the causal mediation analysis. Apathy syndrome was associated with faster impairment of cognition and elevation of the Aβ burden. The effects of apathy on cognitive function and life quality were mediated by Aβ pathology, including CSF Aβ_42_/total tau ratio, and Aβ deposition in the prefrontal regions. Apathy syndrome was the risk factor for cognitive deterioration; meanwhile, frontal Aβ burden was the risk factor for apathy conversion. Apathy syndrome is an early manifestation of cognitive decline and there are bidirectional roles between apathy syndrome and Aβ pathology. Prefrontal Aβ pathology influenced the pathway from apathy to cognitive decline.

## Introduction

Apathy, defined as a quantitative reduction of goal-directed activity compared to the patient’s previous level of functioning [1], is the most prevalent behavioral and psychological syndrome in up to 88% of patients with Alzheimer’s disease (AD) and the vast majority of frontotemporal dementia [2]. The syndrome persists for at least 4 weeks and affects at least two of the three apathy dimensions, including behavior/cognition, emotion, and social interaction [1]. The occurrence of apathy is associated with a faster cognitive decline for patients and a higher burden for caregivers [3], which causes the isolation and drastic daily routine alterations of families with dementia patients. Although neuropsychiatric symptoms have long been recognized as emerging after dementia, recent reports have found apathy may proceed with cognitive decline, up to 5 years prior to the manifestation of the cognitive convention [3]. Therefore, understanding the effects and pathology of apathy in cognitive decline is necessary to measure dementia progression and explore therapy strategy.

In 2016, a new A/T/N classification scheme based on biomarkers was proposed for making an early AD diagnosis. As cerebrospinal fluid (CSF) biomarkers, Aβ_42_ stands for amyloid pathology, phosphorylated (p-tau) stands for tau pathology, and total tau (t-tau) stands for neurodegeneration [4]. Few studies had explored the neuropathologic correlations of neuropsychiatric syndromes and apathy in particular [5]. Existing research suggested that AD-type pathology might be a determinant of apathy. Small sample studies revealed that apathy might be correlated with Aβ burden and cognitive decline in Parkinson’s disease [6], AD [3, 7], and non-dementia elderly [8, 9]. Conflicting results reported that apathy scores positively correlated with CSF t-tau and p-tau levels[10], but not Aβ_42_ [11], or noncorrelation with CSF AD biomarkers [12].

To this day, it is still disputable how apathy syndrome impacts cognitive decline and whether AD-type biomarkers modulate the relationship of apathy with cognitive function. Thus, the present study aimed to examine the effect of apathy on cognitive functions and AD-type biomarkers. In addition, we tested whether the influences of apathy on cognition were mediated by AD pathology.

## Materials and methods

### Subjects

The data of 2272 adults were downloaded from the Alzheimer’s Disease Neuroimaging Initiative (ADNI) database (adni.loni.usc.edu) [13, 14]. A total of 1215 entries were excluded; 949 subjects with missing data, including CSF AD biomarkers, cognitive assessments, and *APOE* genotype, 239 subjects diagnosed with dementia, and 27 subjects with outliers of AD biomarkers. A total of 1057 non-dementia elderly were eventually involved in this study. At baseline, all the subjects received cognitive assessments and CSF tests. Meanwhile, the AV45 positron emission tomography (PET) data from 663 individuals were downloaded. After 2 years, 774 individuals were willing to be re-assessed, 342 received CSF tests, and 413 were scanned by AV45 PET. In ADNI, cognitively normal subjects had Mini-Mental State Examination (MMSE) scores between 24 and 30, a clinical dementia rating (CDR) of 0, no memory complaints, and Geriatric Depression Scale-15 (GDS-15) score < 7. Mild cognitive impairment (MCI) subjects were diagnosed according to the criteria of Petersen et al. [13], which included MMSE scores between 24 and 30, a CDR of 0.5, a GDS-15 score of <7, a memory complaint verified by an informant, and objective evidence of memory loss. Through follow-ups, dementia patients were diagnosed with an MMSE score between 20 and 26, a CDR of 0.5–1, and a GDS-15 score of <7.

ADNI is a multi-site data set designed to test the clinical symptoms, imaging, genetic, and biochemical biomarkers of AD, launched in 2003. Data collection and sharing in ADNI were approved by institutional review boards of all participating institutions and written informed consent was obtained from all participants or their guardians in accordance with the Declaration of Helsinki. The participants are older adults aged 55–90 years. Each participant underwent an in-person interview for health and neuropsychological assessments at baseline and annual follow-up.

### Measures

#### Neuropsychological and neuropsychiatric assessments

Neuropsychological tests included the following measures: global cognition by MMSE, Alzheimer’s Disease Assessment Scale-cognitive sections, CDR, life quality by Functional Activities Questionnaire (FAQ), and depression screening by GDS-15.

Neuropsychiatric symptoms were assessed with the Neuropsychiatric Inventory (NPI), which is an informant-based instrument, measuring the presence (0 = absent, 1 = present), frequency, and severity (1 = mild, 2 = moderate, and 3 = severe) of multiple symptoms including delusions, hallucinations, apathy, agitation, depression, and so on. We used the presence vs. absence of apathy as our dichotomous indicator and divided all subjects into apathy positive (+) and apathy negative (−) groups [15]. The severity ratings had three levels as follows: mild rating defined that the apathy was perceptible but not obvious, moderate rating defined as obvious but not very prominent, and severe rating defined as very prominent change. The questionnaire about apathy absence included eight questions as follows:

1. Does the participant seem less spontaneous and less active than usual?
2. Is the participant less likely to initiate a conversation?
3. Is the participant less affectionate or lacking in emotions compared to his/her usual self?
4. Does the participant contribute less to household chores?
5. Does the participant seem less interested in the activities and plans of others?
6. Has the participant lost interest in friends and family members?
7. Is the participant less enthusiastic about his/her usual interests?
8. Does the participant show any other signs that he/she doesn’t care about doing new things?

#### CSF AD-type biomarkers

Before analysis, concentrations were all normalized into *Z*-score and outliers beyond ±3δ were excluded (*n* = 27). All 1057 subjects had CSF AD biomarkers, including Aβ_42_, p-tau, and t-tau proteins. The ADNI used the fully automated and highly standardized Roche Elecsys immunoassay to assess AD biomarkers.

All subjects were also binarized into Aβ negative (−) or positive (+) based on whether their CSF Aβ_42_ was normal or abnormal. Aβ+ individuals had a CSF Aβ_42_ < 976.6 pg/ml [16]. T-tau and p-tau were expressed in ratio to Aβ_42_, because they were reported as better predictors of Aβ deposition and cognitive decline [17].

#### *APOE* genotype

DNA was extracted with the QIAamp®DNA Blood Mini Kit and amplified by PCR with forward primers 14 5′-ACGGCTGTCCAAGGAGCTG-3′ (rs429358) and 5′-CTCCGCGATGCCGATGAC-3′ 15 (rs7412). *APOE* genotype was performed through restriction fragment length polymorphism technology.

#### Regional Aβ PET data

All image acquisition procedures were described in detail on the ADNI website (http://adni.loni.usc.edu/methods/documents/). Briefly, Aβ PET images were acquired in four frames of 5 min each, 50–70 min p.i. for ^18^F-florbetapir and 90–110 min p.i. for ^18^F-florbetaben. Regional Aβ PET data were downloaded from the ADNI Laboratory of Neuro Imaging database (adni.loni.usc.edu/methods/pet-analysis). Standardized uptake value ratios (SUVR) were computed using the whole cerebellum as a reference region. Through the Cox proportional hazards model, we selected the following regions of interest: the medial orbitofrontal cortex (mOFC) and the pars orbitalis cortex (POC).

### Statistical analyses

Based on the presence of apathy, all the subjects were binarized into apathy positive (+) and apathy negative (−) groups. The data were not normally distributed; therefore, statistical significance was assessed using nonparametric tests. The Mann–Whitney *U*-test was used for continuous variables and the *χ*^2^-test was used for categorical variables to test the differences between two groups. Statistical power was calculated using the PASS software. The alternative hypothesis of two means unequal was adopted with a simulation of 1000.

The linear mixed-effect model depicted the longitudinal effects of apathy on the clinical outcome differences in the two groups. Furthermore, we also depicted the longitudinal difference of apathy severity between the Aβ positive (+) and Aβ negative (−) groups. The model included random slope and intercept terms for each participant. Age, education years, sex, and *APOE4* genotype were included as covariates. Intracranial volume (ICV) was also adopted as a covariable in the imaging analysis. A Kaplan–Meier plot was constructed to assess the risk of cognitive conversion. A time-dependent Cox proportional hazards model was run to predict the cognitive conversion and apathy conversion. The cognitive conversion was defined as: (1) MCI progressing into dementia and (2) cognitively normal progressing into MCI or dementia. Apathy conversion was defined as apathy negative (−) at baseline progressing into apathy positive (+) during follow-up. Age, education years, sex, *APOE4* genotype status, and with or without ICV were included as covariates. Smoothing splines were used to establish fitting curves between conversion stages and multiple biomarkers. The stage of change was calculated as the months away from the onset point of conversion and absolute values of different biomarkers were normalized into *Z*-score. The change trends of MMSE, Aβ_42_, and Aβ_42_/t-tau ratio were the opposites of the other biomarkers, so their negative forms were adopted.

Mediation analysis was used to explore whether Aβ pathology biomarkers mediated the causal pathway from apathy to cognitive decline. We assigned *X* to be apathy status (apathy severity at baseline), *M* as the potential mediators (CSF Aβ_42_/t-tau ratio, frontal lobe Aβ SUVR, mOFC Aβ SUVR, and POC Aβ SUVR), and *Y* as the outcome (cognitive function and life quality at 2-year follow-up). In this context, we interpreted the total effect as the amplitude of apathy severity in cognitive decline, both directly and through Aβ pathology biomarkers intermediates. To decompose the total effect into the part explained by Aβ pathology biomarkers and the part due to other factors, we analyzed direct and indirect effects by fitting two models: a mediator and an outcome model. The causal association was observed for a possible mediator and we estimated the causal effects of Aβ pathology biomarkers on the cognitive outcome. Age, education years, sex, and *APOE4* genotype were included as covariates with or without ICV. This analysis was performed to estimate the total effect, direct effect, indirect effect, and their 95% confidence intervals using the PROCESS macro for SPASS [18] with bootstrapping of 1000 iterations. When the total effect and Sobel test were both significant, the mediation effect was considered to exist.

The statistical significance of all tests was set at a two-sided *p*-value < 0.05. All analyses were performed using SPSS 17.0. GraphPad Prism and R version 4.0.3 were used for figure preparation.

## Results

### Participants characteristics

Participant characteristics in ADNI were summarized in Table 1. A total of 1057 individuals without dementia were included in the present study. The participants were in their late midlife (aged 72.72 ± 7.04), with moderate years of education (mean = 16.33 years), and cognitively unimpaired (mean MMSE score = 28.31). Male participants accounted for 53.1%.

**Table 1:** Characteristics of participants.

Characteristics	Total	Apathy (−)	Apathy (+)	*p*/FDR *p* (statistical power)
Baseline				
*N*	1057	943	114	/
Age (years)	72.72 ± 7.04	72.71 ± 7.03	72.82 ± 7.17	0.805
Gender (male%)	53.10	50.40	75.40	<0.001 (1.000)
Education (years)	16.33 ± 2.62	16.36 ± 2.62	16.11 ± 2.64	0.363
Hypertension (%)	41.60	40.90	47.40	0.188
Diabetes (%)	0.50	0.50	0.00	0.436
APOE (ε4%)	45.20	44.00	55.30	0.023 (1.000)
MMSE	28.31 ± 1.75	28.38 ± 1.71	27.85 ± 1.94	0.003 (0.753)
ADAS-cog	14.07 ± 6.74	13.68 ± 6.59	17.26 ± 7.14	<0.001 (0.997)
CDR	0.26 ± 0.25	0.24 ± 0.25	0.41 ± 0.21	<0.001 (1.000)
FAQ	1.97 ± 3.56	1.52 ± 2.98	5.73 ± 5.33	<0.001 (1.000)
CSF biomarkers (pg/ml)				
*N*	1057	943	114	/
Aβ_42_	1002.70 ± 463.73	1014.18 ± 465.90	907.68 ± 435.76	0.017 (0.673)
t-tau	258.21 ± 108.99	257.18 ± 108.47	266.71 ± 113.33	0.392
p-tau	24.62 ± 12.32	24.48 ± 12.25	35.78 ± 12.85	0.281
Regional brain Aβ burden (SUVR)				
*N*	663	569	78	/
Frontal lobe	1.21 ± 0.23	1.22 ± 0.23	1.23 ± 0.24	0.975
Left mOFC	1.19 ± 0.26	1.19 ± 0.25	1.22 ± 0.30	1.805
Right POC	1.20 ± 0.24	1.29 ± 0.24	1.22 ± 0.28	2.041
2-Year follow-up				
*N*	774	698	76	/
MMSE	27.48 ± 2.97	27.56 ± 2.94	26.68 ± 3.14	0.009 (0.625)
ADAS-cog	14.92 ± 9.38	14.41 ± 9.17	19.63 ± 10.02	<0.001 (0.988)
CDR	0.36 ± 0.34	0.33 ± 0.33	0.63 ± 0.31	<0.001 (1.000)
FAQ	3.74 ± 6.32	3.17 ± 5.79	8.97 ± 8.34	<0.001 (1.000)
CSF biomarkers (pg/ml)				
*N*	342	306	36	/
CSF Aβ_42_	940.65 ± 449.72	956.29 ± 456.17	807.73 ± 369.94	0.053
CSF t-tau	275.48 ± 118.14	273.42 ± 117.75	292.91 ± 121.71	0.293
CSF p-tau	26.13 ± 13.15	25.83 ± 13.00	28.70 ± 14.25	0.199
Regional brain Aβ burden (SUVR)				
*N*	413	369	44	/
Frontal lobe	1.22 ± 0.25	1.21 ± 0.24	1.31 ± 0.29	0.062
Left mOFC	1.21 ± 0.27	1.20 ± 0.26	1.31 ± 0.32	0.041 (0.470)
Right POC	1.20 ± 0.25	1.19 ± 0.24	1.29 ± 0.32	0.131

*Aβ* amyloid β, *ADAS* Alzheimer’s Disease Assessment Scale, *CDR* Clinical Dementia Rating, *CSF* cerebrospinal fluid, *FAQ* Functional Activities Questionnaire, *MMS* Mini-Mental State Examination, *mOFC* medial orbitofrontal cortex, *p-tau* phosphor-tau, *POC* pars orbitalis cortex, *SUVR* standardized uptake value ratios, *t-tau* total tau.

**Fig. 1:**
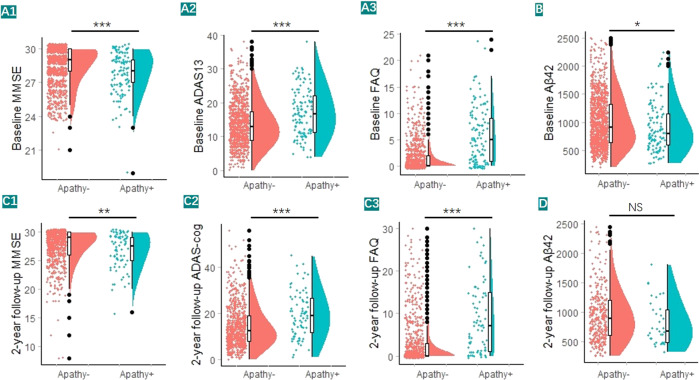
Associations of apathy with clinical outcomes, including cognitive functions, quality life, and CSF Aβ_42_ level. We categorized the total sample into apathy− subgroup and apathy+ subgroup. **A1**, **A2**, **C1**, **C2** Lower cognitive functions were significant in apathy+ individuals than their normal counterparts. **A3**, **C3** Lower quality of life were significant in apathy+ individuals. **B**, **D** Higher CSF Aβ_42_ (low statistical power) were significant in apathy+ individuals. Dots represented individuals’ clinical outcomes, with violin plots showing their median values and distributions. *p < 0.05, ***p < 0.001.

Through NPI assessment, individuals were divided into two groups (943 apathy− and 114 apathy+) and 663 participants underwent brain Aβ PET scanning (569 apathy− and 78 apathy+). Participants with apathy tended to be male and *APOE* ε4 carrier. They also had a more impaired cognitive function and life quality (Fig. 1A1–A3) and higher cerebral Aβ burden (with low statistical power) (Table 1 and Fig. 1B). After 2 years, 774 individuals (698 apathy− and 76 apathy+) received neuropsychological assessments, 342 individuals (306 apathy− and 36 apathy+) received CSF AD biomarkers testing, and 413 individuals (369 apathy− and 44 apathy+) underwent Aβ PET scanning. The analyses of the 2-year follow-up were almost consistent with the result at baseline (Fig. 1C1–C3, D). Due to the large difference in sample size between groups, statistical power was calculated for statically significant analysis. At baseline, the statistical powers of the variables were all above 0.75, except CSF Aβ_42_ level (0.673); however, at 2-year follow-up, MMSE and Left mOFC Aβ SUVR were only 0.625–0.470, which was due to the small sample size.

### Longitudinal effects of apathy on cognitive function, CSF Aβ level, and regional Aβ burden

**Fig. 2:**
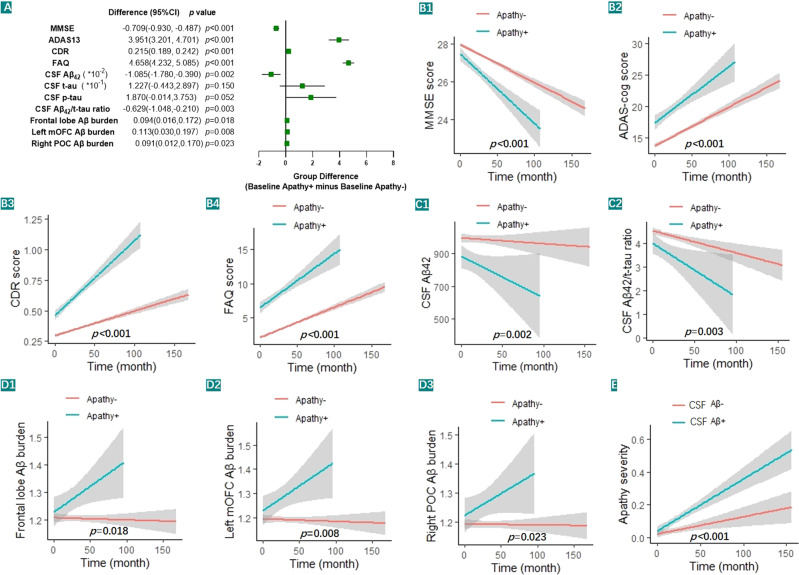
Changes in clinical outcomes affected by apathy syndrome or Aβ levels. **A** Clinical outcomes between apathy- and apathy+ groups based on the linear mixed-effects model after adjusting for age, education years, sex, APOE ε4 status, and ICV (when imaging analysis). **B1**–**B4** The apathy+ group had a lower cognitive function and life quality than the apathy− group. **C1**, **C2** The apathy+ group had decreased CSF Aβ levels than the apathy− group. **D1**–**D3** The apathy+ group had a higher regional Aβ burden. **E** The Aβ+ group had a faster apathy severity elevation than the Aβ− group.

A total of 1057 individuals underwent an in-person interview at baseline and annual follow-up, and the follow-up time was up to 156 months (*n* = 5465 person-times in total). The longitudinal effects were used to explore the effects of apathy on cognitive functions, CSF Aβ_42_ level, and regional brain Aβ deposition. We found that individuals with apathy displayed faster cognition and life quality impairments (Fig. 2A, B1–B4) and faster elevation of CSF Aβ burden (Fig. 2A, C1, C2) after controlling the age, education years, sex, and *APOE* ε4 status. Moreover, individuals with apathy showed faster Aβ deposition in cortical regions including the frontal lobe, left mOFC, and right POC after controlling the age, education years, sex, *APOE* ε4 status, and ICV (Fig. 2A, D1–D3). Compared with the 468 Aβ− elderly, the 589 Aβ+ elderly showed faster elevation of apathy severity (*β* = 0.083, *p* < 0.0001, Fig. 2E), after controlling the age, education years, sex, and *APOE* ε4 status (*n* = 2008 person-times in total).

A total of 68 cortexes, IVC, age, education years, sex, and *APOE* ε4 status were included as covariables to identify risk regions of Aβ deposition for apathy conversion through the Cox proportional hazards model. We found that the left mOFC (hazard ratio (HR) = 840.409, 95% confidence interval (95% CI) = 1.867–378243.514, *p* = 0.031) and the right POC (HR = 85.113, 95% CI = 1.511–4782.305, *p* = 0.031) were the risk regions.

### Causal mediation analyses

**Fig. 3:**
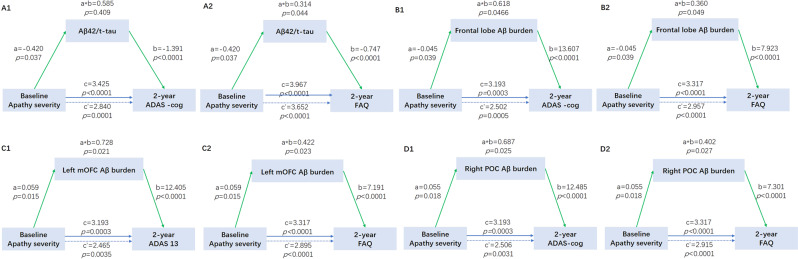
Mediation effects of Aβ pathology on the association of apathy severity with 2-year follow-up cognitive outcomes. Aβ pathology included the CSF Aβ/t-tau ratio (**A1**, **A2**), frontal lobe Aβ SUVR (**B1**, **B2**), left mOFC Aβ SUVR (**C1**, **C2**), and right POC Aβ SUVR (**D1**, **D2**). Blue lines showed the total effect (c) of apathy on 2-year follow-up cognitive functions, blue dotted lines showed the direct effect (c’), and green lines depicted the mediation effect (a*b) of Aβ pathology. Path weights were only shown for significant paths and were expressed as effect and *p*-value.

We investigated whether apathy severity contributed to cognitive impairments via modulating Aβ pathology. We did not find the mediation pathway of Aβ_42_, t-tau, or p-tau alone from apathy to cognitive decline. However, we found the mediation effect of other Aβ pathology biomarkers, including CSF Aβ_42_/t-tau ratio (Fig. 3A1, A2), frontal lobe Aβ burden (Fig. 3B1, B2), left mOFC Aβ burden (Fig. 3C1, C2), and right POC Aβ burden (Fig. 3D1, D2), which mediated the association between apathy severity and cognitive impairment after 2 years (ADAS-13 and FAQ). The effect was considered partial mediation, with the proportion of mediation varying from 7.92% to 22.80%.

### Apathy and cognitive conversion risk

In the cohort, 953 follow-up participants (aged 72.94 ± 7.05, 54.1% males with follow-up duration: 43.52 ± 31.84 months, maximum = 156 months) were included for exploring the incident cognitive decline. Among them, 69 cognitively normal subjects developed MCI or dementia, 204 MCI subjects developed dementia, and 680 participants had censored data. Furthermore, 858 subjects with apathy, aged 72.92 ± 7.01 and 51.5% males, were included to explore the apathy conversion risk during the follow-up. Among them, 227 subjects without apathy at baseline developed apathy syndrome and 631 subjects had censored data.

**Fig. 4:**
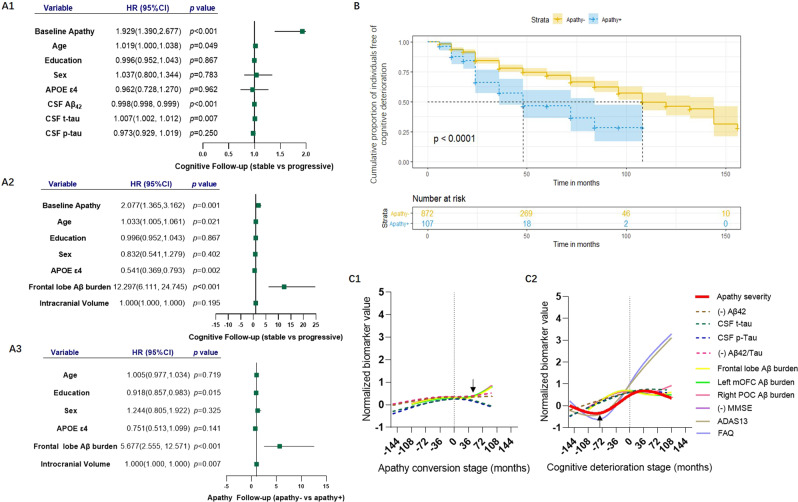
Apathy risks for cognitive conversion and the temporal sequence of biomarkers during the process of apathy conversion. **A1**, **A2** Cox proportional hazards model estimated apathy and Aβ pathology risks for cognitive conversion after adjusting for age, education years, sex, APOE ε4 status, and ICV (when imaging analysis). **A3** The frontal Aβ burden was assessed for apathy conversion after adjusting for age, education years, sex, APOE ε4 status, and ICV. **B** Compared to subjects without apathy, those with apathy were associated with a higher risk of cognitive conversion through the Kaplan–Meier curve. **C2** The temporal sequence of biomarkers showed that apathy syndrome began as early as 5–6 years before the onset of cognitive convention. **C1** During the process of apathy conversion, Aβ pathology biomarkers elevated slowly at 10 years before apathy onset and changed markedly at 3 years after apathy.

The results of the Kaplan–Meier analysis and the log-rank test showed a significant difference in the cumulative proportion of individuals free of cognitive deterioration between apathy− and apathy+ individuals (*χ*^2^ = 27.548, *p* < 0.001) (Fig. 4B). Individuals with apathy, higher CSF Aβ_42_ level, or frontal lobe Aβ deposition had an increased risk of cognitive conversion compared to those without apathy through the Cox proportional hazards model with age, education years, sex, *APOE*, and with or without ICV as covariables (Fig. 4A1, A2). Moreover, subjects with higher frontal Aβ deposition had a higher risk for apathy conversion with age, education years, sex, *APOE*, and ICV as covariables (Fig. 4A3).

### The temporal course of biomarkers

A smoothing spline was used to establish fitting curves to indicate the temporal course of different biomarkers during cognitive decline and apathy conversion. A total of 69 NC that developed MCI or dementia and 204 MCI that developed dementia were included for analysis of cognitive deterioration, whereas 114 apathy+ at baseline and 227 apathy− developing into apathy+ during follow-up were included for analysis of apathy conversion.

“0” on the X-axis indicated the onset point of conversion and values on both sides of “0” indicated the months away from the conversion point. During the process of cognitive decline, AD pathology biomarkers preceded the other biomarkers. Apathy syndrome began almost simultaneously with the turning point of cognitive tests, as early as about 5–6 years before cognitive conversion (Fig. 4C2). During the process of apathy conversion, Aβ pathology biomarkers elevated slowly at 10 years before apathy onset and changed markedly 3 years after apathy (Fig. 4C1).

## Discussion

The present study had three main findings. First, apathy syndrome could significantly elevate the risk of cognitive decline and brain Aβ burden. Second, the influences of apathy severity on cognition and life quality were associated with Aβ pathology in the prefrontal regions. Third, apathy syndrome began 5–6 years before cognitive conversion and the brain Aβ burden elevated slowly up to 10 years before the onset of apathy.

Our results were consistent with previous longitudinal findings that demonstrated apathy syndrome predicting cognitive conversion. A study of 332 MCI elderly over 3 years showed a higher incidence of dementia with HR of 1.62 in subjects with apathy [19] and a study of 873 elderly over 2 years found that apathy was associated with incident cognitive decline [20]. Similarly, in the present study, a larger and longer follow-up cohort, we found non-dementia subjects with apathy had a higher risk of cognitive conversion with HR of 1.93–2.08. In addition, our analysis revealed that apathy predicted a higher cerebral Aβ burden, which aligned with some previous studies [8, 9], despite other negative findings [11, 12]. The first postmortem study of AD that explored apathy pathology nearly 30 years ago showed a combination of apathy with increased neurofibrillary tangle burden [21]. A study of 157 non-dementia subjects followed for up to 4 years found that apathy correlated with Aβ burden through ^18^F-flutemetamol PET [8]. Another study of 413 participants on CSF examination followed up for 3 years found a significant correlation between lower Aβ_42_ and greater rate of apathy [18]. Lanctôt et al. [22] thought the association with p-tau burden was more consistent across all stages of AD and Aβ burden was associated with apathy earlier in the disease process. Our study found that apathy subjects had a higher brain Aβ burden during the longitudinal observation. Apathy syndrome and brain Aβ burden, including CSF Aβ_42_/t-tau ratio and frontal lobe Aβ deposition, were risk factors for cognitive decline. Moreover, frontal lobe Aβ deposition manifested to be the risk factor for apathy conversion and Aβ-positive individuals showed greater apathy severity through the longitudinal analysis results. All the above findings suggested the bidirectional roles of apathy and Aβ pathology.

Our mediation analysis showed that apathy severity led to cognitive impairment at a 2-year follow-up through the mediation effects of brain Aβ burden. First, CSF Aβ_42_/t-tau ratio and Aβ deposition in the prefrontal regions, including the left mOFC and right POC, could play the mediation role. Gad A found that Aβ burden in the cortical regions modified the association between cognitive impairment and p-tau, and the association was stronger in individuals with greater Aβ burden [18]. Although we did not find the effects of CSF p-tau in the present study, further tau PET analysis might provide more information. Second, we found the effect of apathy at the 2-year follow-up cognitive function but not at baseline cognitive function, which suggested that the influence of apathy syndrome was long term. During the progress of cognitive deterioration, CSF Aβ decreased first, followed by cortical Aβ deposition, CSF p-tau elevation, Fluro-deoxyglucose PET decrease, hippocampus atrophy, and cognitive impairment in order [23], which could explain the delayed and long-term effects induced by Aβ pathology. The effects of medication induced by Aβ pathology varied from 7.92% to 22.8%. In addition, by analyzing the temporal course of biomarkers, apathy syndrome began 5–6 years before cognitive conversion. This finding was similar to a small sample study that included 76 healthy elderly with a mean age of 69.9 years. It found that apathy scores and rates increased over 5 years, and apathy changes were associated with informant ratings of cognitive decline in the years prior to baseline assessment [24].

The mechanism by which Aβ pathology is involved in apathy contributing to cognitive impairment remains unclear. Some researchers thought that cored amyloid plaques damaged the dopamine transporter and caused impaired motivation [25], and others found apathy occurred due to lesions affecting the medial and orbital parts of the prefrontal cortex [26]. Previous findings showed that atrophy of mOFC and white matter abnormalities within mOFC were associated with apathy [27, 28]. In the present study, we found that Aβ deposition in the prefrontal regions, including the left mOFC and right POC, began up to 10 years before apathy onset through a time-biomarker fitting curve and was also a risk factor for apathy conversion through Cox proportional hazards analysis. These findings suggested that these two prefrontal regions with high Aβ burdens were involved in the mechanism of apathy. Although there was no report about POC in apathy, atrophy [28] and dysconnectivity [29] in pars orbitalis gyrus were found in cognitively deteriorated patients with schizophrenia and attention-deficit/hyperactivity disorder [30].

Moreover, amyloid pathology was found to play a mediation role in associating minimal depressive symptoms with cognitive impairments in the non-dementia population [31]. Lower Aβ_42_ and higher p-tau were also confirmed to be related to an increased probability of depression and apathy over time [32]. There was an overlap between apathy and depression, which shared common clinical features in AD. Diminished interest, psychomotor retardation, fatigue/hypersomnia, and lack of insight were similar in both syndromes. However, symptoms such as dysphoria, suicidal ideation, self-criticism, hopelessness, and pessimism were unique to depression [22]. The similar symptoms of apathy and depression could be due to the same neuropathology pathway, and accurate diagnostic strategy and longitudinal observation could give more distinguishment and deeper insight.

There are limitations to this study. The studied sample was restricted to those with apathy syndrome. The statistical power of some variables was <0.75 at baseline and 2-year follow-up, which was due to the small sample size. The effect size of apathy might be underestimated given the low incident rate of apathy in non-dementia population. Aβ PET analysis was adopted, but not tau PET imaging, which weakened the exploration of tau pathology in the mechanism of apathy. Cognitively normal and MCI subjects were included in this study, which might have affected the population heterogeneity bias. As a theoretical statistical analysis, the final fact about the mediation effect of Aβ pathology on the association between apathy and cognitive decline still needs to be verified by multi-dimensional analysis in the future study. NPI-apathy scale has certain weakness such as data being acquired from the informant, which might bring the recall bias.

In summary, this study indicated that apathy syndrome was an early manifestation of cognitive decline, which could help define high-risk populations suitable for early prevention of dementia. There were bidirectional roles between apathy syndrome and Aβ pathology, and prefrontal Aβ burden influenced the pathway from apathy to cognitive decline.

Data collection and sharing in ADNI were approved by institutional review boards of all the participating institutions in accordance with the Declaration of Helsinki.

## Data Availability

A complete listing of ADNI investigators can be found at: http://adni.loni.usc.edu/wpcontent/uploads/how_to_apply/ADNI_Acknow-ledgement_List.pdf.
